# Rowan’s Rugby Fatality Prompts Canada’s First Concussion Legislation

**DOI:** 10.1017/cjn.2019.15

**Published:** 2019-05

**Authors:** Charles Tator, Jill Starkes, Gillian Dolansky, Julie Quet, Jean Michaud, Michael Vassilyadi

**Affiliations:** Canadian Concussion Centre, Toronto Western Hospital, Toronto, Ontario, Canada; University of Ottawa Faculty of Medicine, Ottawa, Ontario, Canada; Memorial University of Newfoundland Faculty of Medicine, St. John’s, Newfoundland, Canada; Dalhousie University Faculty of Medicine, Halifax, Nova Scotia, Canada; Children’s Hospital of Eastern Ontario, Ottawa, Ontario, Canada

**Keywords:** Concussion, Athletes, Traumatic brain injury, Second impact syndrome

## Introduction

Concussions are a major public health problem in Canada because there are so many, not every concussed person recovers, and the consequences of concussion such as second impact syndrome (SIS) can be severe, even fatal,^1^ as in the case of Rowan Stringer whose case is described in the Brief Communication in this issue of the journal. She was 17 when she died of SIS in July, 2013 after a brain injury sustained in high school rugby. The treating physicians and surgeons were uncertain of the exact mechanism of her death. The case was remarkable because evidence of SIS was discovered by the inquest held to determine the exact cause of death.

### The Rowan Stringer Inquest

In October, 2013, one of the authors (CHT) recommended an inquest into the death of Rowan Stringer. The request was made to the Regional Supervising Coroner in Ottawa, Dr. Louise McNaughton-Filion. CHT spoke to Rowan’s parents who agreed that an inquest should be called “if it could possibly help someone else”. Dr. McNaughton-Filion became the inquest’s presiding coroner, Mr. Mark Moors was the inquest’s Crown Attorney, and Detective Sameer Sharma, City of Ottawa Police, was assigned as well as five jurors. CHT was appointed Expert Witness to assist the inquest in determining the cause of death and to advise about measures to prevent death on the pitch. CHT received all the records, conferred with officials, provided a report to the coroner and jury, and was a witness at the Ottawa inquest which lasted from May 9 to June 3, 2015.

The inquest successfully determined the cause of death based on evidence obtained from many witnesses who knew Rowan and knew the events prior to her death, including her parents, teachers, friends, rugby coaches, referees, and rugby league officials. Evidence was provided by the health care professionals who treated her including authors MV who performed the neurosurgical procedures and JM who performed the neuropathological examination. The most revealing evidence was Rowan’s texted messages to and from her friends during the 5 days before her fatal injury obtained by Detective Sharma who retrieved Rowan’s cell phone, downloaded all the texted messages, and had them transcribed. She texted about the blows to the head she sustained during a rugby game on May 3, 2013 and then again 3 days later on May 6, 2013 in a rugby game when she was “kicked in the head.” The symptoms she texted including headache, fatigue, and tinnitus made it highly likely that she sustained concussions on May 3 and 6. The final fatal brain injury occurred on May 8, 2013 after which she never regained consciousness. Between May 3 and 8, no adult diagnosed a concussion or was told about her May 3 and 6 brain injuries. She played while still symptomatic from the previous injuries. She did not reveal her symptoms to her parents or other adults in those 5 days. Rowan also texted her friends that she had “googled concussion” and surmised that she “probably had a concussion.”

### The Inquest Report and Its Effects

On June 3, 2015, the inquest jury concluded that Rowan died of SIS. The inquest provided the evidence of the antecedent brain injuries and the jury clearly concluded that the cause of death was “Malignant Cerebral Edema due to Second Impact Syndrome, due to Traumatic Brain Injury”.^2^ A recent review of SIS identified 17 cases (all male athletes, predominantly in early adolescence, 10 participating in American football and 7 in other sports including hockey) that met the following criteria: observed second head impact with immediate neurological deterioration; cerebral edema that could not fully be explained by structural pathology; verification of continuous post-concussive symptoms after the first impact up to the time of the second; and evaluation by a trained medical professional after the observed first impact.^3^ Rowan Stringer certainly met the first three criteria for SIS, but could not have met the fourth because she did not reveal her symptoms and did not consult a medical professional. Thus, this review supports the accuracy of the inquest jury’s conclusion that she had SIS.

The jury also made 49 recommendations to prevent athletes from dying from the consequences of concussions.^2^ It is unknown how many athletes die of undiagnosed SIS because the evidence of antecedent concussion is missing if the concussed person does not reveal the symptoms and they are not apparent to team officials, family, or friends because of lack of awareness of the condition. SIS is difficult to treat but is preventable if the concussion is recognized and leads to medical examination and advice to desist from play until symptoms completely disappear. Thus, PLAYERS MUST SPEAK and inform school and league officials and their teachers, coaches, referees, and parents. It is now acknowledged based on clinical experience that the recently concussed brain is more vulnerable to subsequent injury if a second brain injury occurs prior to full clinical recovery from the first injury. The inquest jurors were well informed and their 49 recommendations were designed to prevent other athletes from getting SIS. The recommendations were made to Coroner McNauhton-Filion for consideration for implementation by the three involved Ontario Ministries: Tourism, Culture and Sport; Education; and Health and Long-term Care. The first recommendation was the following: That the Government of Ontario adopt an Act (“Rowan’s Law”) governing all youth sport, both school-based and non-school-based, and that the Act should recognize the importance of four criteria in protecting children and youth: (1) providing education on sport-related concussions to athletes, coaches, and parents; (2) removing a child or youth athlete from play if a concussion is suspected; (3) ensuring the child or youth does not return to play until he or she has received medical clearance; and (4) ensuring appropriate return-to-learn and return-to-play strategies are in place.^2^

## Canadian Measures to Improve Concussion Management

Recently, there have been many noteworthy efforts by several Canadian national and provincial organizations and individual Canadian researchers and policy makers to develop new guidelines for concussion diagnosis and management, including return to play, return to school/learn, and return to work. In 2015, the Government of Canada through the Public Health Agency of Canada initiated the “harmonization” of concussion management in all sports. Parachute Canada, the country’s national injury prevention agency, was commissioned, and in 2017, the “Canadian Concussion Guidelines” were completed and distributed^4^ representing the world’s first national sports concussion guideline. After recognition and diagnosis of concussion, safe management involves graduated return to play, and no return to full contact game play in collision sports until all symptoms have returned to pre-injury levels. The diagnosis of concussion should be made by a medical doctor or nurse practitioner, and all concussed athletes should be cleared by a medical doctor or nurse practitioner before returning to play. These are the hallmarks of concussion management clearly enunciated in Parachute’s Canadian Concussion Guidelines.^4^ The Guidelines underline the importance of early detection of all brain injuries including intracranial blood clots in all age groups and diffuse brain swelling in children/adolescents.

### Canadian Experience with SIS and Current Criteria

The only published case of SIS in Canadian sports was reported in 1968 in a New Brunswick hockey player, age 16, who fell striking his head on the ice. Four days later, he was body-checked and again fell striking his head on the ice. He died the same day and autopsy showed a swollen brain with brainstem hemorrhages and herniation at the foramen magnum, neuropathological evidence of 4-day-old contusions and a small amount of subdural and subarachnoid blood.^5^ Rowan Stringer is likely the first case of SIS in whom the diagnosis was established by the deceased’s texted messages. Indeed, it is the authors’ view that coroners’ inquests should be considered in all sports injury deaths in youngsters and young adults when there is uncertainty about the exact cause of death. It is highly likely that this measure would substantially increase the number of substantiated cases of SIS.

#### The Case for Rowan’s Law Concussion Legislation

Zackery Lystedt at age 13 played high school football in 2006 in the State of Washington. After a hard hit during a game, he continued to play while symptomatic, and then in the same game had a second brain injury thought to be SIS. He was left with severe neurological deficits, and his parents and doctors campaigned for a law to prevent similar catastrophes. The “Lystedt Law” was passed by the State of Washington in 2009 and includes mandatory removal from a game or practice, examination by a health care professional if a concussion is suspected, and mandatory concussion education for players, teachers, coaches, and parents.^6^ By 2014, similar concussion laws had been passed in all 50 US states, but not in any Canadian province. In Ontario, in 2012, Bill 39 advocating concussion legislation received first reading, but the legislature was prorogued shortly after, and the next government did not reintroduce the bill. Many have wondered whether Rowan’s 2013 concussions would have been recognized if Bill 39 had become law in 2012? As an alternative to legislation and recognizing the need to enhance concussion recognition and management, in 2014, the Ontario Ministry of Education introduced a “Policy and Procedure Memorandum” on concussion called PPM158.^7^ Indeed, Ontario became the first province in Canada to adopt regulations about concussion education and management in all public schools. However, PPM158 did not cover private schools, universities, and non-school sport venues and teams.

After the inquest in 2015, Rowan’s parents, Gordon and Kathleen Stringer, joined the campaign for concussion legislation in Ontario. A bill to examine the concept of “Rowan’s Law” was passed in the Ontario Legislature in December, 2015. The government then appointed the Rowan’s Law Advisory Committee with former coroner Dr. Dan Cass, as chair, and one of the authors (CHT) as a member. The Committee met during 2016–2017 and reported in September, 2017. The first recommendation was that Ontario should enact Concussion Legislation to be known as Rowan’s Law.^8^

#### Rowan’s Law Concussion Legislation, Bill 193

Rowan’s Law Concussion legislation was introduced in the Ontario Legislature on December 14, 2017 and became law on March 6, 2018.^9^ The legislation will help prevent concussions and improve the recognition and management of those that occur. The legislation will strengthen the principles of PPM158 by making them apply to all school-based sports in Ontario, both private and public, and hopefully universities, too, and most importantly the legislation will cover all non-school-based sports leagues and venues. Thus, Rowan’s death from SIS will be recognized and memorialized for contributing to concussion prevention and management. Hopefully, all provinces and territories in Canada will enact similar concussion laws and name them “Rowan’s Law” or other appropriate name. Athletes, young and old, will benefit from the advocacy that followed her death. “Rowan’s Law” will ensure improved education about concussion, improved recognition and management of concussion, and enhanced prevention of concussion, all of which will allow players to stay in the game, safely and longer. Most importantly, it will teach kids and youths that players must speak!

## Messages to All Those Involved in Youth Sports

Rowan Stringer’s death proves that fatal malignant brain swelling can occur after a concussion and supports ongoing efforts to protect young athletes including women from ‘second hits’. Distinct from other cases, Rowan was a late adolescent female and was playing rugby. The revelation of antecedent injuries and symptoms through the injured person’s texted messages uncovered by the inquest is unique and emphasizes the importance of injury prevention measures. For our team of authors, this point was the crux of the tragedy for this patient and her family. It is important to note that neuropathology alone cannot confirm SIS. It is imperative that physicians knowledgeable about concussions including pediatricians, emergency medicine specialists, family doctors, sports medicine physicians, neurologists, neurosurgeons, and other health care professionals should continue efforts to educate the public about concussions and other brain injuries. Rowan Stringer’s unfortunate outcome highlights the need to expand knowledge about how to prevent initial and repeated sports-related brain injuries. While scientific and medical knowledge related to the pathophysiology of concussion improves, translation of that knowledge to players, coaches, teachers, and parents must be ensured. For example, ongoing evidence-based review of return-to-play guidelines^4^ and plans to convey that material to pediatric athletes and those supervising their play is essential.
